# Light green background enhances reading performance in visual display terminal tasks

**DOI:** 10.3389/fpsyg.2025.1627013

**Published:** 2025-07-25

**Authors:** Xiangyun Li, Yu Guan, Ruilin Wu, Xuejun Bai

**Affiliations:** ^1^Key Research Base of Humanities and Social Sciences of the Ministry of Education, Academy of Psychology and Behavior, Tianjin Normal University, Tianjin, China; ^2^Faculty of Psychology, Tianjin Normal University, Tianjin, China; ^3^Department of Psychology, Beihang University, Beijing, China; ^4^Tianjin Key Laboratory of Student Mental Health and Intelligence Assessment, Tianjin, China

**Keywords:** background color, visual fatigue, emotion, reading performance, visual display terminal

## Abstract

Visual context plays an important role in reading behavior. However, the effects of background color on reading performance remain underexplored. This study investigated how background color (white vs. light green) affects reading performance, visual fatigue, emotion, and physiological responses in first language (L1, Chinese) and second language (L2, English) reading contexts. Forty university students completed reading tasks under both background color conditions, and self-report, behavioral, eye-tracking, and physiological data were collected. The results showed differential effects of background color across L1 and L2 reading contexts. In L1 reading experiment, a light green background significantly increased pupil diameter (indicating lower visual fatigue), reduced negative emotion, and improved reading performance compared to a white background. Moreover, background color significantly affected skin temperature among female participants only. In L2 reading, the green background also increased pupil diameter, though this effect was limited to female readers, with no significant improvements in reading performance or emotional response. These findings suggest that perceptual features of digital reading environments can influence multiple aspects of reading, including visual fatigue, emotion, and performance, with varying effects across L1 and L2 reading contexts. Notably, the effects of background color appear to be moderated by task complexity and gender, with female readers showing greater sensitivity to background color effects, particularly under more demanding reading conditions. The study highlights the role of individual differences and perceptual context in shaping reading experience, with implications for interface design and digital learning platforms.

## 1 Introduction

Digital screens have become the primary medium for reading in modern environments. Visual display terminals (VDTs), including computers, smartphones, tablets, and e-readers, are widely used in daily reading activities (Li et al., 2021; Yin et al., 2022). However, prolonged use of these screens has been linked to visual fatigue and eye discomfort. Some VDT interfaces have attempted to alleviate visual fatigue by enabling users to modify background colors, often offering softer tones such as light green for enhanced visual comfort. Previous studies have shown that colors can affect physiological responses, psychological states, and behaviors (Choi et al., 2020; Gu et al., 2016; Mehta and Zhu, 2009; Savavibool et al., 2018; Sun and Liu, 2016; Wang et al., 2025; Wang et al., 2021). Yet, little is known about how background color specifically affects reading performance on digital screens. Therefore, this study aims to examine the effects of background color on reading performance and explore the potential underlying mechanisms.

Research on the impact of background color on reading performance remains inconclusive. Some studies have found that, compared to white backgrounds, colored backgrounds can significantly improve reading speed in both children with autism and typically developing children (Ludlow et al., 2006; Veszeli and Shepherd, 2019). However, Yamazaki (2010) found that Japanese college students’ performance on English reading tests under light blue and blue background conditions appeared numerically higher than under the white background condition; however, the differences were not statistically significant. It is worth noting that the first two studies employed reading-aloud tasks. Such tasks involve relatively shallow cognitive processing. In contrast, Yamazaki (2010) used a second-language reading comprehension task, which requires deeper processing and imposes a higher cognitive load. This increased cognitive demand may have attenuated the effects of background color. Research on Chinese reading has also yielded inconsistent findings. Lin and Huang (2013) found that reading accuracy was highest with a blue background, followed by green, red, and gray; however, their study excluded a white background. In contrast, other studies reported better reading performance with white backgrounds than with colored ones (An and Li, 2012; Xie et al., 2013). Wang and Chen (2003) revealed that reading accuracy was higher with high-contrast text-background color combinations, such as black text on a white background or blue text on a yellow background. More recently, Guo et al. (2024) found that a green background enhanced the efficiency of Chinese character search in a visual search task. However, contrary to these findings, Wang et al. (2025) reported that background color had no significant effect on reaction time or accuracy in a similar Chinese character search task, further highlighting the inconsistency across studies. The previous research fails to consistently demonstrate the superiority of colored backgrounds over white ones. Moreover, conclusions drawn from studies using high-saturation colors may not generalize to low-saturation hues such as light green. Thus, whether a light green background can enhance reading performance on VDTs compared to a white background remains an open question that warrants further investigation. This question constitutes the first research focus of the present study.

Prolonged exposure to VDT screens can lead to visual fatigue, characterized by eye strain, dryness, blurred vision, and headaches (Blehm et al., 2005; Fan et al., 2024). Light green is often regarded as an eye-friendly background color. It may help reduce visual fatigue and, in turn, enhance reading performance on VDTs. Whether this effect exists remains to be determined. This forms the second research question of the present study. Pupil diameter is used as an objective physiological indicator of visual fatigue, as it tends to decrease with the onset of fatigue (Fan et al., 2024; Li et al., 2004; Murata et al., 2001; Wang et al., 2025). According to Murata et al. (2001), the correlation coefficients between subjective fatigue and pupil diameter ranged from −0.60 to −0.68 (*p*s < 0.01). Previous studies have found that pupil diameter is more sensitive and objective than subjective ratings in evaluating visual fatigue (Jiang et al., 2015). Therefore, pupil diameter is employed as the primary index of visual fatigue in this study.

Color is widely recognized as being closely associated with human emotions (Jonauskaite et al., 2024). This association is theoretically grounded in the Color-in-Context Theory (Elliot, 2015; Elliot and Maier, 2007, 2012; Elliot et al., 2007), which posits that colors can carry specific meanings. These associations between colors and meanings are shaped by both social learning and biological predispositions. When perceiving a color, people tend to retrieve relevant experiences, which in turn elicit distinct emotional reactions. For instance, red is commonly linked to excitement or unpleasantness, whereas blue and green are generally associated with calmness (Elliot, 2019; Elliot et al., 2007; Lu et al., 2018). It has been proposed that the psychological and behavioral effects of color are primarily driven by intrinsic color–meaning associations (Gil and Le Bigot, 2014; Gu et al., 2016). Al-Ayash et al. (2016) found that a blue learning environment made students feel more relaxed and significantly reduced their heart rate compared to red and yellow environments, although no significant differences were observed in reading comprehension scores. Similarly, Lu et al. (2003) reported that middle school students experienced more positive emotions when writing on green paper than on white paper, whereas primary school students showed no such difference. The researchers attributed this finding to middle school students’ greater awareness of their own psychological experiences. Notably, this study did not observe significant gender differences in color perception. However, other studies have indicated gender-based variation. For example, women have been found to exhibit stronger emotional reactions to colors (Jakovljević et al., 2021) and to be more sensitive in distinguishing between blue and green (commonly referred to as “grue”) (Fider and Komarova, 2019). Wu et al. (2022) further found that interface color only affected memory performance in women. Based on these findings, the present study proposes a third research question: Does light green evoke more positive emotions, which in turn enhance reading performance among college students? In addition, it examines whether this effect differs by gender.

In addition, one possible reason for the mixed findings on the effect of background color during reading could be explained by task complexity. In particular, second language (L2) reading is generally more cognitively demanding than first language (L1) reading. For unskilled bilinguals, their two languages share semantic representations and the semantic access of L2 words is mediated by L1 translation equivalents (Kroll and Stewart, 1994; Wang et al., 2023). Drawing on Perceptual Load Theory (Lavie, 1995) and the findings of Yamazaki (2010), it can be inferred that L2 reading imposes greater attentional demands than L1 reading, which may in turn diminish or eliminate the effects of background color. Therefore, the present study will examine the effects of background color separately in Chinese (L1) and English (L2) reading.

Beyond objective indicators such as accuracy rate and reaction time, subjective evaluations can provide valuable complementary information on reading performance. Task Difficulty Rating (TDR), a form of perceived difficulty, is a subjective evaluation of task demands that is influenced by cognitive, motivational, and emotional appraisals. It is partly based on assessments of task complexity and novelty, performance outcomes, and disruptions in cognitive processing or access to required knowledge (Fulmer and Tulis, 2013). Since reading is not only a mechanical decoding process but also involves higher-level comprehension and mental effort, subjective perceptions of difficulty can capture dimensions of reading performance that objective measures alone may overlook. Including TDR alongside traditional performance metrics thus provides a more comprehensive and multidimensional evaluation of reading performance.

In summary, the present study explores how background color influences reading performance and examines the mechanisms behind this effect through two experiments. Experiment 1 will focus on Chinese (L1) reading, while Experiment 2 will examine English (L2) reading. Both will explore the effects of background color. Both experiments will use a 2 (background color: light green vs. white) × 2 (gender: male vs. female) mixed factorial design. Background color will be treated as a within-subjects factor, and gender as a between-subjects factor. The hypotheses are as follows: (1) Background color will influence visual fatigue, emotion, and reading performance. Light green is expected to reduce visual fatigue, increase positive emotion, and improve reading performance. (2) Background color will interact with gender. Females are expected to be more sensitive to color-related effects. (3) The effects of background color on reading performance will be mediated by visual fatigue or emotion. (4) The impact of background color will differ between Chinese and English reading. Stronger effects are expected in Chinese reading.

## 2 Experiment 1: the impact of background color on Chinese reading performance

### 2.1 Methods

#### 2.1.1 Participants

The sample size for the experiment was determined using G*Power 3.1 software (Faul et al., 2007), based on an effect size of *f* = 0.25, *α* = 0.05, and statistical power (1-β) = 0.8, yielding a required sample size of 34 participants. A total of 40 native Chinese-speaking students (20 females, mean age 22.6 years) from one university participated in the experiment. All participants have normal vision (without corrective lenses), no color blindness or color weakness, and are unaware of the purpose of the experiment. Each participant received a compensation of ¥25 upon completion of the study. The study were reviewed and approved by Ethics Committee of Tianjin Normal University. The participants provided their written informed consent to participate in this study.

#### 2.1.2 Design

**Figure 1:**
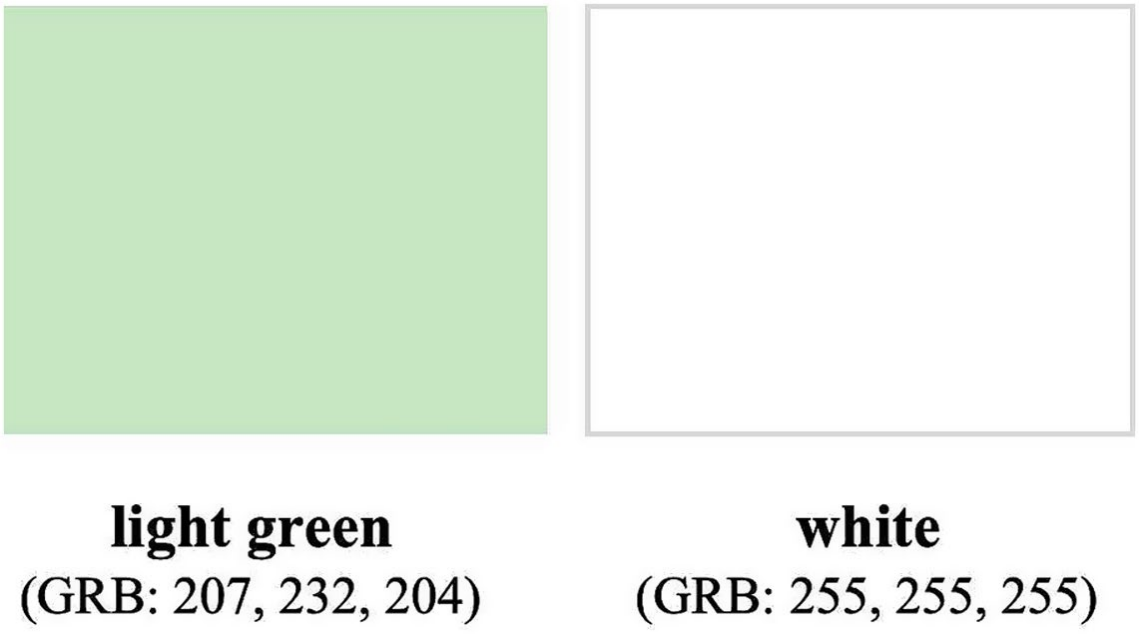
The background colors used in the experiment.

A 2 (background color: light green vs. white) × 2 (gender: male vs. female) mixed factorial design was adopted. Background color will be treated as a within-subjects factor, and gender as a between-subjects factor. The background colors (see Figure 1) used were light green (RGB: 207, 232, 204) and white (RGB: 255, 255, 255).

The dependent variables included: (1) Visual fatigue, measured by pupil diameter; (2) Emotional state, including Positive Affect (PA) score and Negative Affect (NA) score measured by Positive and Negative Affect Schedule (PANAS; Watson et al., 1988); (3) Autonomic nervous system activity, measured by Galvanic Skin Response (GSR), Skin Temperature (SKT), and Heart Rate (HR); (4) Reading performance, assessed by Reaction Time (RT), accuracy, and Task Difficulty Rating (TDR).

#### 2.1.3 Materials

Two equally difficult test sets (Set A and Set B) were developed, each consisting of 20 multiple-choice questions, with four options per question. The process of ensuring equivalent difficulty involved the following steps:

Sixty reading comprehension questions were initially selected from China Civil Service Exam and divided into two sets, each containing 40 questions (including 20 anchor items). To control reading load, the total length of the question stems in both sets was 1,254 characters (excluding anchor items), and all choices consisted of four-character idioms for consistency.

Ninety-two college students with normal or corrected-to-normal vision were randomly selected and divided into two groups (matched for gender, academic level, and major). Each group completed one of the two test sets.

After excluding data from careless responders, 84 valid data points remained. The following procedures were carried out: First, a Classical Test Theory (CTT) analysis was conducted to assess item difficulty (pass rate) and discrimination (point-biserial correlation). Ten items exhibiting extreme difficulty or low discrimination were removed. Second, an Item Response Theory (IRT) analysis was performed using PARSCALE 4.1. Items with low information values were subsequently eliminated. Third, the remaining items were matched in terms of difficulty and discrimination to generate two final test sets.

**Figure 2:**
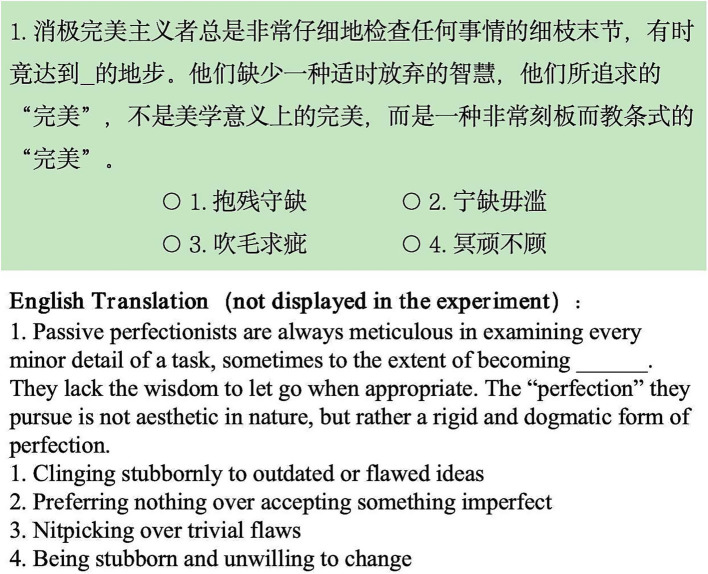
An example of the experimental materials under light green background in Exp 1.

Statistical analysis confirmed that the two sets were equivalent in difficulty, *t*(38) = −0.01, *p* > 0.05. Each final set consisted of 20 questions, with the total number of characters in the question stems being nearly identical: 1,789 in set A and 1,787 in set B. An example of the experimental materials is shown in Figure 2.

#### 2.1.4 Instruments/apparatus

The experimental tasks were presented on a 13-inch ThinkPad E430 laptop using E-Prime 2.0, with a screen resolution of 1,366 × 768 pixels and a brightness level set to 38 cd/m^2^. Participants’ pupil diameter was recorded using a Tobii Glasses portable eye tracker at a sampling rate of 30 Hz.

Participants’ emotion was assessed using the Positive and Negative Affect Schedule (PANAS; Watson et al., 1988). The PANAS consists of two subscales: Positive Affect (PA) and Negative Affect (NA), each containing 10 items. The internal consistency reliability of the PANAS has been reported to range from 0.86 to 0.90 for PA and from 0.84 to 0.87 for NA, indicating good reliability. In the present study (moment instructions), Cronbach’s alpha was 0.89 for the PA subscale and 0.85 for the NA subscale. The PANAS has also demonstrated good convergent validity, with coefficients ranging from 0.89 to 0.95. Participants were instructed to rate the extent to which they experienced each emotion “at the present moment” using a 5-point Likert scale ranging from 1 (not at all) to 5 (extremely).

Previous studies investigating the psychological effects of color have employed physiological indicators reflecting autonomic nervous system activity, such as Galvanic Skin Response (GSR), Skin Temperature (SKT), and Heart Rate (HR) (Al-Ayash et al., 2016; Bai et al., 2016; Hou et al., 2008). Accordingly, in the present study, autonomic nervous system activity was monitored using the Infiniti Multi-Parameter Biofeedback Device, which recorded GSR, SKT, and HR.

#### 2.1.5 Procedure

Before the experiment, participants were seated comfortably while the biofeedback device was attached to their left hand, and the eye tracker was fitted. A 9-point calibration of the eye tracker was conducted, followed by a 3-min baseline measurement of autonomic nervous system activity using the biofeedback device.

After familiarizing themselves with the experimental procedure and completing a practice session, participants began the formal task. They were instructed to read and comprehend each question—presented with the stem and options simultaneously— as quickly and accurately as possible, and to respond by pressing a key (1–4 on the numeric keypad, corresponding to the four options). The next question appeared immediately after a response was made.

Upon completing the task under one background color condition, participants completed the PANAS scale and rated the task difficulty using a 5-point Likert scale (1 = no difficulty, 5 = very difficult). They then took a short break, during which the devices were reconnected and recalibrated, and a new baseline was recorded before proceeding to the second background color condition. The order of background color presentation and the pairing of background colors with test sets were counterbalanced to control for order effects.

The entire experiment lasted approximately 40 min. Following the experiment, a subset of participants was interviewed, with questions primarily focused on whether different background colors had influenced their reading experience. The study was conducted in a quiet, controlled environment during daytime hours to minimize external interference.

### 2.2 Results

Reaction time (RT) was defined as the average time spent reading the material, including reading the question stem and selecting an answer, for correctly answered questions, with extreme values exceeding three standard deviations excluded (accounting for 0.8% of the data). The dependent measure for pupil diameter, GSR, SKT, and HR was the relative change, calculated as the ratio of the mean value during the task to that during the baseline (calibration) period. Due to equipment malfunctions, participants with incomplete or abnormal recordings were excluded, with removal rates ranging from 2.5 to 15%.

A two-factor mixed ANOVA was conducted for each dependent variable using SPSS 27.0, with background color as a within-subjects factor and gender as a between-subjects factor. Descriptive statistics for the different condition are shown in Table 1. In this study, significance levels are indicated as follows: ***p < 0.001, **p < 0.01, *p < 0.05, and underlined *p* < 0.10.

**Table 1:** Means and standard deviations of relevant variables in Exp 1.

Variables		Male		Female	
		White	Light green	White	Light green
Visual fatigue	Pupil diameter	0.94(0.08)	1.01(0.11)	0.96(0.09)	0.99(0.09)
Emotion	PA score	2.82(0.65)	2.72(0.59)	2.32(0.84)	2.24(0.78)
	NA score	1.44(0.58)	1.31(0.34)	1.31(0.40)	1.23(0.39)
Autonomic nervous activity	GSR	1.40(0.46)	1.34(0.33)	1.50(0.74)	1.61(0.94)
	SKT	1.01(0.08)	1.00(0.01)	0.98(0.07)	1.07(0.14)
	HR	1.03(0.07)	1.01(0.06)	1.01(0.06)	1.01(0.05)
Reading performance	RT(s)	18.02(6.37)	17.64(5.81)	18.56(5.26)	17.44(4.89)
	Accuracy	0.68(0.07)	0.66(0.13)	0.67(0.13)	0.68(0.10)
	TDR	2.50(0.83)	2.35(0.88)	2.84(0.83)	2.58(0.84)

For pupil diameter, a significant main effect of background color was observed, *F*(1,30) = 11.95, *p* < 0.01, *η*_p_^2^ = 0.28, with larger pupil diameter under the light green background compared to the white background. No significant main effect of gender or interaction was found, *F*s < 1.39.

For PA score, a significant main effect of gender was found, *F*(1,38) = 4.94, *p* < 0.05, *η*_p_^2^ = 0.11, with males reporting higher PA scores than females. No significant main effect of background color or interaction was found, *F*s < 1.91. For NA score, a significant main effect of background color was found, *F*(1,38) = 4.27, *p* < 0.05, *η*_p_^2^ = 0.10, with lower NA scores under the light green background compared to the white background. No significant main effect of gender or interaction was found, *F*s < 1.

**Figure 3:**
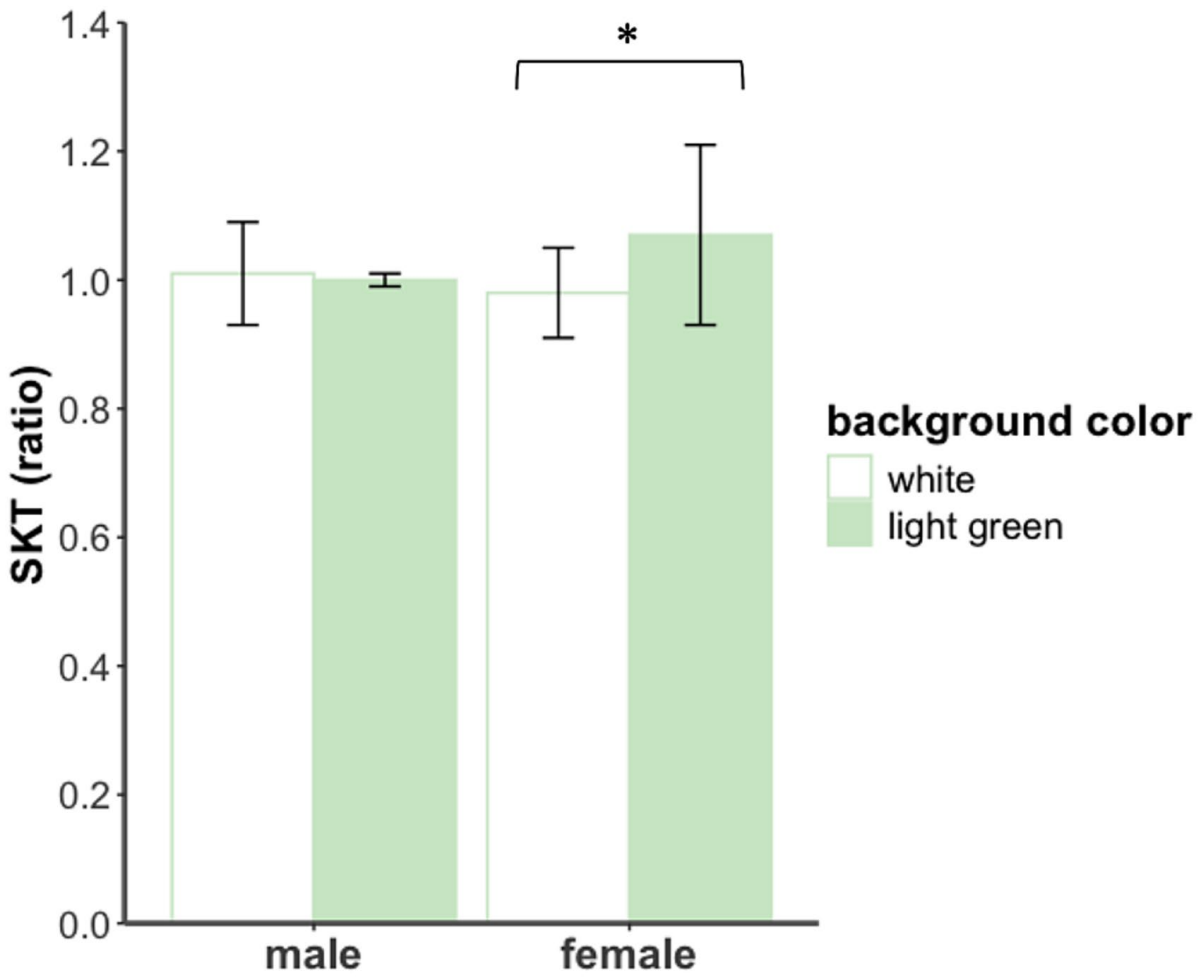
SKT of male and female participants under different background color conditions in Exp 1.

For SKT, no significant main effects were found (*F*s < 3.34). Notably, a significant interaction (see Figure 3) effect was observed, *F*(1,36) = 5.27, *p* < 0.05, *η*_p_^2^ = 0.13. Simple effects analysis revealed that in females, skin temperature was significantly higher under the light green background compared to the white background, *F*(1,19) = 5.58, *p* < 0.05, *η*_p_^2^ = 0.23. In males, the difference was not significant, *F*(1,17) = 0.33, *p* > 0.05. For GSR and HR, no main effects or interactions were found (*F*s < 1.26).

For RT and accuracy, no significant main effects or interactions were found (*F*s < 1.35). For TDR, a main effect of background color was significant, *F*(1,37) = 5.03, *p* < 0.05, *η*_p_^2^ = 0.12, with participants reporting lower task difficulty under the light green background compared to the white background. No significant main effects of gender or interactions were found, *F*s < 1.27.

To further test whether the background color effect on reading performance (indexed by TDR) is mediated by the effect on negative emotion (indexed by NA score) or visual fatigue (indexed by pupil diameter), two separate simple mediation models and one multiple mediation model were conducted using MEMORE macro (version 3.0) for SPSS 27.0, which is specifically designed for mediation analysis in two-condition within-participant designs (Montoya and Hayes, 2017; Wang and Wen, 2018). For all mediation models, percentile bootstrap confidence intervals were generated based on 5,000 bootstrap resamples.

**Figure 4:**
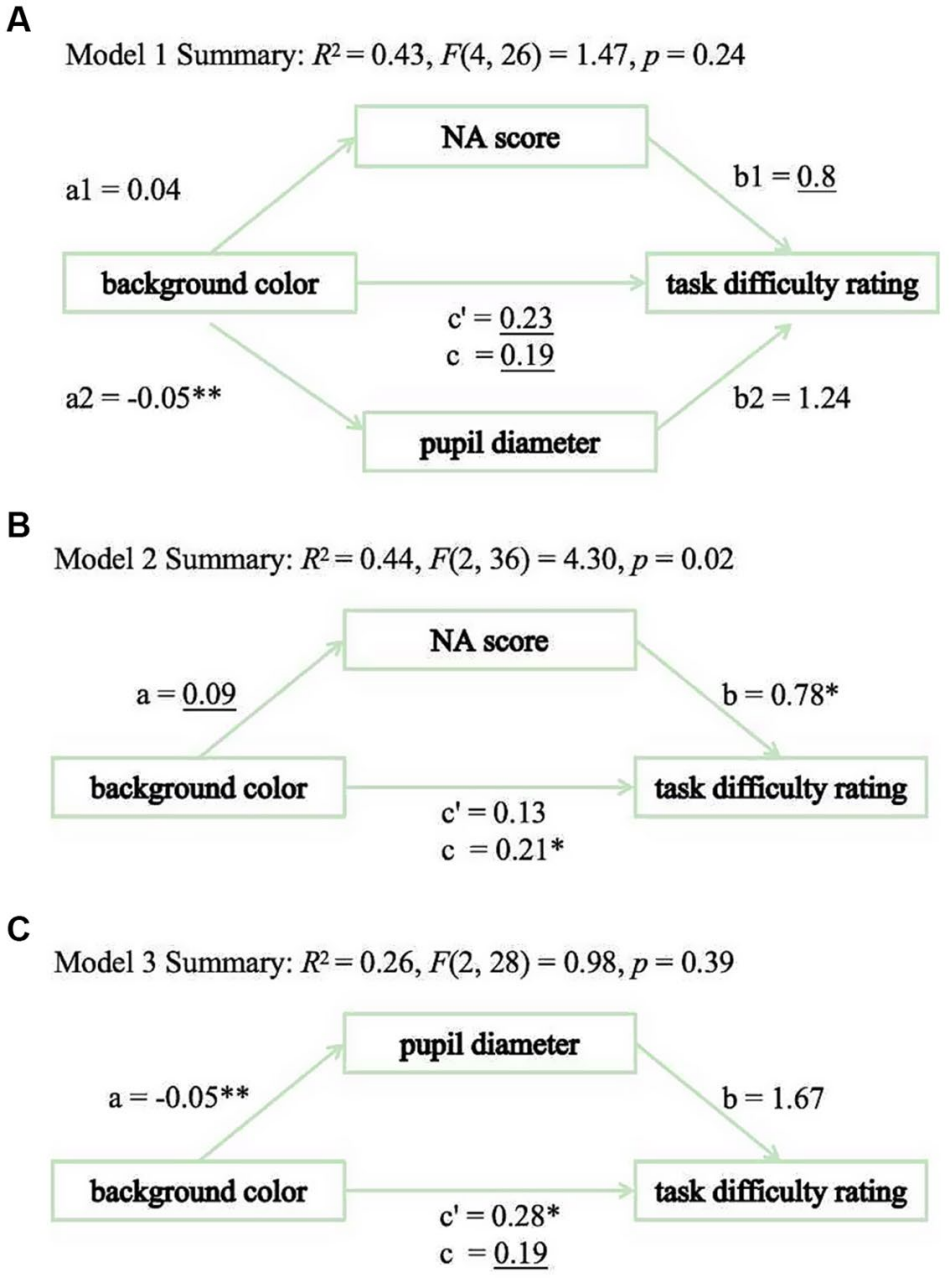
Mediation analysis in Exp 1. **(A)** Model 1. **(B)** Model 2. **(C)** Model 3.

The multiple mediation model (see Figure 4A) revealed non-significant total indirect effect (indirect effect = −0.03, 95% CI = [−0.19, 0.22]), or on-significant indirect effects for both pupil diameter (indirect effect = −0.06, 95% CI = [−0.21, 0.07]) and negative emotion (indirect effect = 0.03, 95% CI = [−0.02, 0.23]). For the simple mediation models, neither the indirect effect through negative emotion (indirect effect = 0.07, 95% CI = [−0.01, 0.27], see Figure 4B) nor pupil diameter (indirect effect = −0.09, 95% CI = [−0.25, 0.08], see Figure 4C) reached statistical significance, as their confidence intervals included zero. Considering the moderating effect of gender on the background color effect, mediation analyses were conducted separately for male and female participants. The results showed that, for both groups, the mediation effects through pupil diameter (male: indirect effect = 0.07, 95% CI = [−0.11, 0.52]; female: indirect effect = −0.19, 95% CI = [−0.39, 0.03]) and NA score (male: indirect effect = 0.05, 95% CI = [−0.07, 0.36]; female: indirect effect = 0.07, 95% CI = [−0.04, 0.40]) were not significant. These results suggest that the effect of background color on reading performance was not clearly mediated by negative emotion or visual fatigue.

## 3 Experiment 2: the impact of background color on English reading performance

Experiment 1 examined the effect of background color on L1 (Chinese) reading performance, showing that a light green background reduced visual fatigue and negative emotion, and enhanced reading performance. Given that second language (L2) reading is more cognitively demanding than L1 reading, Experiment 2 will further investigate the impact of background color on L2 (English) reading performance.

### 3.1 Methods

#### 3.1.1 Participants and design

Same as Experiment 1.

#### 3.1.2 Materials

**Figure 5:**
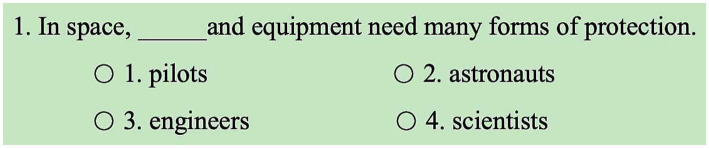
An example of the experimental materials under light green background in Exp 2.

Two sets of English test questions (Set A and Set B) of equivalent difficulty were prepared, each consisting of 20 multiple-choice questions with four answer options. The questions were sourced from the China Graduate Candidate Test, and the difficulty equating process followed the same procedure as described in Experiment 1. An example of the experimental materials is shown in Figure 5.

#### 3.1.3 Instruments and procedure

Same as Experiment 1.

### 3.2 Results

Variables were calculated in the same way as Experiment 1. Similarly, 2 (background color: light green, white) × 2 (gender: male, female) ANOVAs were performed, with background color as a within-subjects factor and gender as a between-subjects factor. Descriptive statistics for the different conditions are shown in Table 2.

**Table 2:** Means and standard deviations of relevant variables in Exp 2.

Variables		Male		Female	
		White	Light green	White	Light green
Visual fatigue	Pupil Diameter	0.95(0.09)	0.97(0.07)	0.96(0.08)	1.03(0.09)
Emotion	PA Score	2.55(0.69)	2.55(0.67)	2.34(0.68)	2.30(0.73)
	NA Score	1.60(0.57)	1.70(0.59)	1.34(0.55)	1.33(0.48)
Autonomic nervous activity	GSR	1.39(0.50)	1.36(0.29)	1.55(0.80)	1.73(0.92)
	SKT	0.99(0.09)	1.00(0.07)	1.01(0.04)	1.00(0.02)
	HR	1.02(0.12)	1.04(0.07)	1.01(0.04)	1.03(0.11)
Reading performance	RT(s)	20.35(5.48)	20.32(6.16)	19.84(4.89)	18.49(5.47)
	Accuracy	0.51(0.15)	0.53(0.18)	0.63(0.14)	0.56(0.12)
	TDR	3.05(0.71)	3.26(0.65)	2.89(0.99)	2.89(0.66)

**Figure 6:**
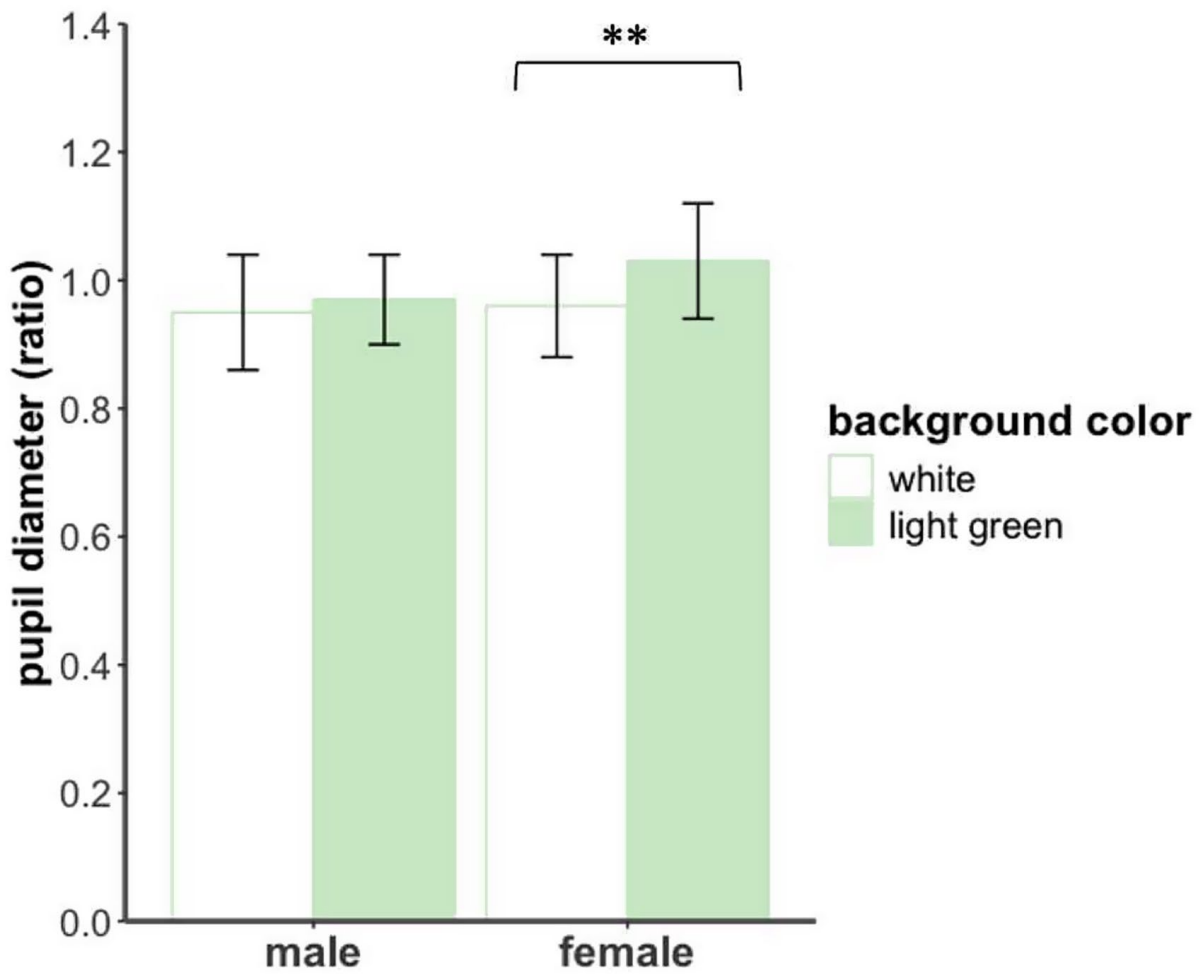
Pupil diameter of male and female participants under different background color conditions in Exp 2.

For pupil diameter, a significant main effect of background color was observed, *F*(1,30) = 13.74, *p* < 0.001, *η*_p_^2^ = 0.31, with larger pupil diameters under the light green background compared to the white background. The main effect of gender was not significant, *F*(1,30) = 1.15, *p* > 0.05. Notably, the interaction effect was significant (see Figure 6), *F*(1,30) = 4.49, *p* < 0.05, *η*_p_^2^ = 0.13. Simple effects analysis revealed that in the female group, pupil diameter was significantly larger under the light green background than under the white background, *F*(1,15) = 14.48, *p* < 0.01, *η*_p_^2^ = 0.49. In contrast, no significant difference was found in the male group, *F*(1, 15) = 1.52, *p* > 0.05.

No significant main effects or interactions were found for any of the remaining variables, including PA, NA, GSR, SKT, HR, RT, accuracy, and TDR (*F*s < 3.86).

## 4 Joint analyses of experiments 1 and 2

Since the two experiments involved similar reading tasks—one in Chinese and the other in English—joint analyses were conducted to further examine how task moderates the background color effect. A 2 (task: Chinese, English) × 2 (background color: light green, white) × 2 (gender: male, female) ANOVA was used to analyze data across both experiments. Given our primary interest in the effect of task and its interactions, we report only the relevant results here.

A significant main effect of task was found for NA score [*F*(1,38) = 6.69, *p* < 0.05, *η*_p_^2^ = 0.15], RT [*F*(1,34) = 6.05, *p* < 0.05, *η*_p_^2^ = 0.15], accuracy [*F*(1,34) = 27.70, *p* < 0.001, *η*_p_^2^ = 0.45] and TDR [*F*(1,36) = 17.81, *p* < 0.001, *η*_p_^2^ = 0.33]. Specifically, under the English reading condition, participants showed higher NA scores, longer response times, lower accuracy, and higher TDR, suggesting that English reading was more cognitively demanding than Chinese reading.

A significant or marginally significant interaction between task and background color was found for NA score [*F*(1,38) = 4.25, *p* < 0.05, *η*_p_^2^ = 0.10] and TDR [*F*(1,36) = 3.03, *p* = 0.09, *η*_p_^2^ = 0.08], indicating that the effect of background color depends on the task. Under the Chinese reading condition, a light green background reduced negative emotion and perceived task difficulty compared to a white background (for details, see the Results section of Experiment 1). In contrast, no significant background color effect was found under the English reading condition (for details, see the Results section of Experiment 2).

A three-way interaction among task, background color, and gender was significant for SKT [*F*(1,36) = 4.49, *p* < 0.05, *η*_p_^2^ = 0.11] and pupil diameter [*F*(1,25) = 4.87, *p* < 0.05, *η*_p_^2^ = 0.16], suggesting that the influence of background color was moderated not only by task complexity but also by gender, and that the nature of gender differences varied across tasks.

Additionally, the joint analysis revealed a marginally significant effect of background color on RT [*F*(1,34) = 2.94, *p* < 0.1, *η*_p_^2^ = 0.08]. Participants exhibited a trend toward faster responses under the light green background compared to the white background, suggesting a possible subtle benefit of background color for reading efficiency.

## 5 General discussion

This study examined the impact of background color on reading performance in screen-based contexts. The findings revealed that a light green background significantly alleviated visual fatigue, reduced negative emotion, and improved reading performance. These results underscore the multifaceted influence of background color on users, encompassing visual comfort, emotional regulation, and cognitive performance during digital reading activities.

In Experiment 1, which employed a Chinese reading task, several important findings emerged. First, pupil diameter was significantly larger under the light green background than under the white background. As pupil dilation is commonly associated with reduced visual fatigue in reading contexts, this finding supports Hypothesis 1. Second, the light green background significantly reduced negative emotion, also supporting Hypothesis 1. This result is consistent with previous studies demonstrating that the color green is associated with more positive emotions (Lu et al., 2003; Lu et al., 2018; Gil and Le Bigot, 2014). These results align with the Color-in-Context Theory (Elliot, 2015; Elliot and Maier, 2007, 2012), which posits that the psychological effects of color can be shaped by learned associations. In daily contexts, green is commonly associated with nature, vitality, and pleasant experiences, which may help explain the reduced negative affect observed under the light green condition. Third, background color selectively influenced the autonomic nervous activity of female readers. Specifically, the light green background significantly increased skin temperature in female participants, relative to the white background. This finding supports Hypothesis 2, which predicted that the effect of background color would be moderated by gender. Prior research suggests that skin temperature is positively correlated with emotional valence. Higher emotional valence is typically associated with increased temperature (Bai et al., 2016). This further supports the claim that a light green background reduces negative emotion. Fourth, participants reading materials of equal difficulty under different background conditions reported different task difficulties. Those in the light green condition reported lower perceived task difficulty than those in the white background condition. This result supports Hypothesis 1, indicating that background color can improve reading performance. Fifth, unexpectedly, mediation analyses did not provide evidence that either negative emotion (as indexed by NA score) or visual fatigue (as indexed by pupil diameter) mediated the effect of background color on reading performance (as indexed by TDR). In other words, while the light green background improved reading outcomes, the hypothesized mediating roles of emotion and visual fatigue were not statistically supported. Therefore, Hypothesis 3 was not confirmed. One possible explanation is that certain methodological limitations may have weakened the detection of mediation effects. For example, participants’ emotional states were not measured prior to the experiment, and the emotional valence of the reading materials was not evaluated. These factors may have introduced variability in emotional responses, thereby reducing the sensitivity of the mediation model. Additionally, it is possible that background color affects reading performance through alternative pathways not examined in the current study, such as motivation, attitude, or perceived task engagement (Mehta and Zhu, 2009; Sun and Liu, 2016). These findings highlight the complexity of color’s influence on reading behavior. Future studies should address these methodological issues and consider broader sets of potential mediators to better understand the mechanisms underlying the background color effect.

In Experiment 2, which involved an English reading task, two primary findings emerged. First, compared to the white background, the light green background significantly increased pupil diameter, which further supports the eye-protection hypothesis of light green backgrounds. Notably, this effect was observed only among female participants, lending additional support to Hypothesis 2, which proposed that women are more sensitive to the influence of background color. Second, in contrast to the results observed in Experiment 1, no significant main effects of background color were found on emotional state, autonomic nervous activity, or reading performance. These findings suggest that the impact of background color may vary across different language contexts, and that its effects are more pronounced when reading in one’s native language.

A comparison of the two experiments revealed consistent findings regarding the effect of background color on visual fatigue. The light green background was effective in both the Chinese and English reading tasks. It was associated with increased pupil diameter and reduced visual fatigue. These results support the idea that green backgrounds have an eye-protective effect. Post-experiment interviews provided further support for this finding. Several participants reported that the white background caused visual discomfort, including double vision, glare, and eye fatigue. In contrast, the light green background was seen as more comfortable and less straining on the eyes. However, in the English reading task, the effect of background color reached significance only among female participants, indicating a potential gender difference in sensitivity to background color. This is consistent with previous findings suggesting that women tend to be more sensitive to color stimuli (Fider and Komarova, 2019).

In contrast to the findings on visual fatigue, the two experiments yielded inconsistent results regarding the impact of background color on emotional state and reading performance. This supports Hypothesis 4. Specifically, unlike in the Chinese reading task, background color had no significant effects on emotional state, autonomic nervous activity, or reading performance during English reading. This discrepancy may reflect the increased cognitive demands associated with L2 reading. In L2 reading, readers must access the conceptual representations of English words via the lexical representations of their Chinese translations (Wang et al., 2023). Drawing on Perceptual Load Theory (Lavie, 1995) and findings from Yamazaki (2010), it can be inferred that L2 reading places greater demands on attentional resources compared to L1 reading. As a result, readers may allocate fewer cognitive resources to non-task-relevant stimuli such as background color, thereby reducing its influence during L2 reading tasks. This was also supported by the joint analysis, which showed that task type moderates the background color effect.

Previous studies have reported a white background advantage in reading tasks (An and Li, 2012; Xie et al., 2013). However, findings from the Chinese reading experiment in the present study suggest that a light green background is more conducive to reading comprehension than a white background. This inconsistency may be attributed to several factors. First, the studies differed in their experimental designs. Prior studies employed between-subjects designs, which are more susceptible to individual differences. In contrast, the present study used a within-subjects design for background color. This allowed for better control of participant-related variance. Second, the manipulation of background color varied across studies. Color may exert a variety of psychological effects. In addition to emotion and visual fatigue, which were the primary focus of the present study, color may also influence other cognitive and psychological processes. These include perceptual clarity, attentional allocation, and subjective preferences such as motivation and attitude, as suggested by some researchers (Mehta and Zhu, 2009; Sun and Liu, 2016). For example, red has been shown to activate avoidance motivation and enhance performance on attention-demanding tasks. In contrast, blue tends to activate approach motivation and facilitate creative thinking (Mehta and Zhu, 2009; Sun and Liu, 2016). These potential mechanisms were not examined in the current study and warrant further investigation in future research. Third, there may be differences in color familiarity. Unlike previous studies that used high-saturation colors (e.g., red, blue, yellow, green), the light green background used in this study is relatively common and familiar in daily visual environments. This may lead to greater comfort and reduced distraction.

Previous research suggests that green backgrounds evoke positive emotions (Lu et al., 2003), and that females are more sensitive to color (Jakovljević et al., 2021; Fider and Komarova, 2019). In this study, however, no background color effect or interaction was observed in terms of positive affect, except for a gender difference, where males scored higher. While this may seem inconsistent with prior studies, it is important to note that the emotional measurement tools used in this study differ from those in previous research. For example, Lu et al. (2003) found that green text evoked more positive psychological experiences among middle school students. Green was perceived as softer, more comfortable, aesthetically pleasing, and inducing a calmer, more relaxed feeling. This type of emotional experience more closely corresponds to the dimension measured by the Negative Affect (NA) scale in this study. Therefore, this study found that the green background elicited fewer negative emotions, which is consistent with the findings of Lu et al. (2003). Additionally, in physiological measures, differences were observed only in the female group, where a higher skin temperature under the green background indicated a better emotional experience (Bai et al., 2016), supporting the idea that females are more sensitive to color. Unlike Lu et al. (2003), who did not find a moderating role of gender in middle school students, the results of this study suggest that gender differences become more pronounced with age.

The Chinese reading experiment did not reveal significant effects of background color on reaction time or accuracy. However, it is important to note that this result is specific to the experimental conditions of this study, which may have been influenced by several factors.

First, reading comprehension is a complex cognitive process. The influence of background color may be reduced in such high-level tasks (Liu et al., 2010). Numerous other variables—such as material difficulty—can influence performance outcomes. High difficulty levels, in particular, may result in floor effects, masking any subtle impact of background color. For example, Liu et al. (2010) found that text color had a more pronounced effect on easier questions. Thus, future studies may need to adjust task difficulty to better capture the effects of background color.

Second, as a non-target stimulus, background color likely influences cognitive performance via an accumulative process. Given the short experimental duration in this study, there may not have been sufficient exposure time for the effect to manifest in reaction time or accuracy. This interpretation is supported by earlier findings. These studies suggest that exposure time plays a key role in color-related effects (Al-Ayash et al., 2016). Interestingly, although background color did not significantly influence reaction time in either individual experiment, a combined analysis revealed a trend toward faster responses under the light green condition. This suggests that background color may have a subtle, cumulative effect on reading performance that becomes more detectable after prolonged exposure. In individual tasks, this effect may have been masked due to limited exposure time. Rather than contradicting the individual findings, this trend complements them and provides additional insight into the potential role of background color in enhancing reading efficiency. Future studies should consider the possibility of such cumulative effects when designing experiments.

Finally, writing habits and familiarity may also play a role. In traditional writing and reading habits, black text on a white background is considered conventional and formal. As a result, participants may be more attuned to white backgrounds and less responsive to alternative color conditions. Future research could explore methods to control for such familiarity effects, such as through training, adaptation periods, or pre-exposure paradigms.

Collectively, these factors highlight the complexity of isolating background color effects in reading tasks and underscore the importance of more refined experimental control in future studies.

As discussed in the previous sections, this study may have two potential limitations. First, participants’ emotional states were not measured prior to the experiment, and the emotional valence of the reading materials was not evaluated. Second, the relatively short exposure duration to background color may have limited the detection of its effects. These factors may have reduced the sensitivity of the measures or the ability to detect subtle effects. Future research could address these issues by incorporating baseline emotion assessments, standardized valence ratings, and longer exposure periods.

In summary, this study examined the effects of commonly used “eye-protecting” background colors in VDT interfaces. The results showed that background color can influence individuals in multiple ways. It affects visual comfort, emotional state, and reading performance. Although the two pathways (emotion and visual fatigue) examined in this study were not supported, the findings further suggest that background color may exert its effects through other mechanisms. This calls for further research to explore these possibilities. Importantly, these findings advance our understanding of color effects in digital media and offer practical guidance for the design of digital reading environments, particularly in educational, office, and e-learning contexts.

## 6 Conclusion

Under the conditions of this study, the following conclusions can be drawn: (1) The effects of background color are multifaceted, influencing visual fatigue, emotional state, and reading performance during VDT-based reading tasks. (2) Female readers appear to be more sensitive to background color effects in certain aspects, indicating a potential gender difference in susceptibility. (3) L1 (Chinese) reading is more susceptible to background color effects than L2 (English) reading, particularly in terms of emotion and reading performance.

## Data Availability

The original contributions presented in the study are included in the article/supplementary material, further inquiries can be directed to the corresponding authors.
